# Characteristics of long COVID and the impact of COVID-19 vaccination on long COVID 2 years following COVID-19 infection: prospective cohort study

**DOI:** 10.1038/s41598-023-50024-4

**Published:** 2024-01-09

**Authors:** Yoonjung Kim, Sohyun Bae, Hyun-Ha Chang, Shin-Woo Kim

**Affiliations:** Division of Infectious Diseases, Department of Internal Medicine, School of Medicine, Kyungpook National University Hospital, Kyungpook National University, 130, Dongdeok-ro, Jung-gu, Daegu, 41944 Republic of Korea

**Keywords:** Infectious diseases, Outcomes research

## Abstract

This prospective cohort study aimed to identify characteristics of long COVID and any potential mitigating effects of COVID-19 vaccinations in patients 24 months following COVID-19 infection. Adult patients diagnosed with COVID-19 between February 17, 2020, and March 24, 2020, were scheduled to visit the study hospital four times (6, 12, 18, and 24 months after infection) to assess their symptoms, quality of life, and mental health. Among the 235 patients, 121 (51.5%) completed the study visits. Of these, 59.5% were female, with a median age of 52 years. Mild to moderate disease severity were identified in 101 (83.4%) patients. A total of 75 participants (62.0%) were still experiencing long COVID symptoms 24 months after acute infection. Fatigue, amnesia, difficulty concentrating, and insomnia were the most common symptoms. The frequency of neuropsychiatric symptoms did not differ based on vaccination status or the number of doses received. Quality of life improved over time for the participants, but 32.2% of respondents still reported anxiety/depression at the end of the study. Overall, our cohort demonstrates that long COVID can persist up to 24 months after COVID-19 infection, affecting mental health and quality of life.

## Introduction

The number of severe acute respiratory syndrome-coronavirus-2 (SARS-CoV-2) infections is increasing globally, raising concerns about the ongoing COVID-19 pandemic. Long COVID, a multisystemic condition^1^ that can occur following SARS-CoV-2 infection, is characterized by fatigue, impaired physical and cognitive performance, headache, breathlessness, and other symptoms that interfere with daily activities^2,3^.

While long COVID typically improves over time^4^, neuropsychological symptoms may persist longer than others, increasing the risk of long-term neuropsychological disease development^5^. Potential mechanisms of long COVID include immune dysregulation, autoimmunity, and viral persistence, including neurotropism of SARS-CoV-2^6–8^. Studies on COVID-19-infected mice have reported decreased neuronal generation in the hippocampus, increased cerebrospinal fluid levels of multiple cytokines and chemokines, and increased microglial activation than that in uninfected mice^9,10^.

The frequency and burden of long COVID increase with the severity of acute disease^11^, but it also occurs in patients with mild symptoms who do not require hospitalization^12^. Long COVID has been shown to negatively impact quality of life^13,14^ and can persist for a long time after infection^15^. Investigations into the long-term effects of COVID-19 beyond 2 years are limited. A systematic review of the effect of COVID-19 vaccination on long COVID suggested the possibility of protective and therapeutic effects on long COVID^16^. Furthermore, vaccination before^17–19^ or after^20^ COVID-19 infection has been reported to reduce the risk of long COVID. To date, 70.6% of the world’s population has received at least one dose of a COVID-19 vaccine, with this number up to 88% in the Korean population. In Korea, the percentages of the population receiving second, third, and fourth COVID-19 vaccinations were 87%, 66%, and 15%, respectively^21^. However, no long-term follow-up studies that included vaccination history have been conducted on long COVID 2 years after acute COVID-19 infection. Therefore, this study aimed to investigate the long-term impact of COVID-19 on patients with a confirmed vaccination history and to establish improved treatment plans for patients with long COVID in the future. Long-term studies are necessary to better understand the effects of COVID-19 beyond the first 2 years.

## Methods

### Study participants and design

This prospective cohort study enrolled adult patients in Korea with polymerase chain reaction-confirmed COVID-19 infection between February 17, 2020, and March 24, 2020, and data were collected between August 31, 2020, and March 29, 2022. Initially, 5,252 adult patients with COVID-19 infection were identified from the Daegu Center for Infectious Disease Control and Prevention registry in Daegu and contacted individually by mobile phone. After excluding deceased patients, we included those who agreed to participate and were able to visit the study hospital. Patients were recruited to be evenly distributed by age to reduce age-related bias.

Participants visited Kyungpook National University Hospital four times in the 24 months following the onset or diagnosis of COVID-19. Enrolled patients were those who consented to participate and completed all four hospital visits. A survey was conducted using a modified version of the International Severe Acute Respiratory and Emerging Infection Consortium protocol, translated into Korean^22^ (Supplementary Table S1). Long COVID was defined by 38 symptoms: fever, chills, myalgia, arthralgia, fatigue, cough, sore throat, rhinorrhea, sputum production, dyspnea, palpitations, arrhythmia, chest discomfort, headache, dizziness, cognitive dysfunction, difficulty concentrating, amnesia, abnormal directional sensibility, seizure, paresthesia, globus pharyngeus, hallucination, insomnia, social phobia, depression, anxiety, obsessive thinking, anorexia, diarrhea, nausea or vomiting, anosmia, ageusia, tinnitus, alopecia, skin rashes, pruritis, and COVID toes.

The survey included questions on the following: sex, birth date, COVID-19 diagnosis date, COVID-19 symptom onset date, height, weight, smoking history, quarantine site during acute COVID-19 infection, oxygen treatment history including ventilator usage, extracorporeal membrane oxygenation (ECMO), dialysis at the time of hospitalization, intensive care unit admission history, COVID-19 vaccination history, underlying diseases, symptoms or diseases newly identified after COVID-19 infection, hospitalization history after acute COVID-19 infection, and COVID-19 reinfection history. Disease severity during acute COVID-19 infection was classified into five categories ranging from asymptomatic to critical illness. Clinical data, including symptoms and disease severity during acute COVID-19 infection, was confirmed using the Daegu Center for Infectious Disease Control and Prevention registry.

### Definition

Long COVID was defined as having at least one newly identified intermittent or continuous symptom 3 months after the initial SARS-CoV-2 infection, lasting for at least 2 months, with no other explanation^23^. Vaccination was considered complete in patients after at least (a) 2 weeks after receiving the second dose in a two-dose COVID-19 vaccine series or (b) 2 weeks after receiving a single dose COVID-19 vaccine.

### Outcome measures

The study investigated the clinical characteristics of long COVID and the impact of vaccination on long COVID symptoms, focusing on quality of life and mental health using several scales. These included the EuroQol 5-dimension 5-level (EQ5D) tool, Korean version of the Patient Health Questionnaire-9 (PHQ-9), the Generalized Anxiety Disorder-7 (GAD-7) scale, and the Post-Traumatic Stress Disorder (PTSD) Checklist-5-Korean version (PCL-5-K) scores. The EQ5D score comprises five categories: mobility, self-care, usual activities, pain/discomfort, and anxiety/depression. Each category has five levels to indicate the severity of problems (none, slight, moderate, severe, and extreme). Respondents indicated their health status by selecting the most appropriate statement for each category. The scores for the five categories were then combined to form a five-digit number representing the respondent’s health status^24^. PHQ-9 is an instrument for screening, diagnosing, monitoring, and measuring the severity of depression. PHQ-9 scores were rated using a four-point Likert scale ranging from 0 (not at all) to 3 (nearly every day). Total score can range from 0 to 27, with high scores indicating more severe depression. Based on the original validation studies, the total score is then interpreted to represent no (0–4), mild (5–9), moderate (10–14), moderately severe (15–19), or severe (20–27) depression. A cutoff score of 10 suggests a possible diagnosis of depressive disorder^25^. The GAD-7 is a seven-item self-reported instrument with each item scored on a four-point Likert scale indicating symptom frequency, ranging from 0 (not at all) to 3 (nearly every day). Total scores represent none/minimal (0–5), mild (6–10), moderate (11–15), and severe (> 16) anxiety symptoms. The GAD-7 score can range from 0 to 21, with a score ≥ 10 indicative of generalized anxiety disorder^25^. The PCL-5-K consists of five, single-factor items scored dichotomously as either “yes” (1 point) or “no” (0 points). Higher scores indicate more severe symptoms, and 3 is the cutoff score for significant PTSD^26^. In addition to using these standardized scales, we also evaluated other changes in lifestyle habits potentially related to long COVID.

### Statistical analyses

Descriptive statistics were used to assess demographic differences. Other categorical and noncategorical variables were compared using Fisher’s exact test, chi-square test, Student’s *t*-test, or Mann–Whitney *U* test as appropriate. Clinical characteristics were compared between the symptomatic group and asymptomatic group at 24 months following acute COVID-19 infection to identify the factors affecting the development of long COVID. The frequency of each long COVID symptom was calculated at 6, 12, 18, and 24 months following acute COVID-19 infection and shown as a percentage of the respondents. In addition, we conducted univariate analysis to identify the impact of vaccination on long COVID symptoms. The score distributions from PHQ-9, GAD-7, and PCL-5-K scales were compared between 12-month and 24-month timepoints after acute infection to identify the long-term impact of COVID-19 on psychiatric symptoms. The PHQ, GAD-7, and PCL-5-K scores at 12, 18, and 24 months after acute COVID-19 infection were analyzed with respect to disease severity, using violin plots to show the distribution and peak of the scores from each scale. Furthermore, Sankey flow diagrams were generated to identify changes in the distribution and interaction of major long COVID symptoms over time. For all tests, differences were considered statistically significant at *P* < 0.05. R Statistics version 4.0.2 was used for all statistical analyses (The R Foundation; https://www.r-project.org).

### Ethics approval

This study was reviewed and approved by the Institutional Review Board of Kyungpook National University Hospital (approval no.: 2021–02-003). All methods were performed in accordance with the relevant guidelines and regulations by including a statement in the methods section. All respondents provided digital informed consent before the questionnaire was administered.

## Results

### Participant demographics and characteristics

A total of 235 patients returned to the study hospital for follow-up 6 months after the onset of COVID 19-related symptoms or diagnosis, with 170, 154, and 133 patients visiting at 12, 18, and 24 months, respectively. Among these, 121 patients (51.5%) completed all four visits. The median age of patients was 52 years (interquartile range [IQR] 39.0–61.0 years). A total of 113 patients (93.4%) received complete vaccination after acute COVID-19 infection. Reinfection with COVID-19 was confirmed in five patients (4.1%; Table 1). None of the patients who completed the study had required ECMO or emergency hemodialysis during hospitalization for acute COVID-19, and none were readmitted for further treatment after initial discharge. Additionally, no patients with underlying hematologic malignancies were identified in the cohort. The group with persistent long COVID at 24 months had a higher proportion of women and a lower median height than the group without symptoms at that time point (*P* = 0.02 and *P* = 0.03, respectively). Between the patients who had residual symptoms at 24 months and those who did not, no significant differences were observed in overall age distribution (*P* = 0.42), age at time of COVID-19 infection (*P* = 0.07), body mass index (*P* = 0.40), maximum disease severity during acute COVID-19 infection (*P* = 0.17), newly diagnosed disease after COVID-19 infection (*P* = 0.97), post-infection COVID-19 vaccination status (*P* = 1.00), COVID-19 reinfection (*P* = 0.13), or pre-existing comorbidity (*P* > 0.05 for all conditions queried; Table 1 and Supplementary Table S2).

**Table 1:** Clinical characteristics of 121 cohort patients.

Characteristics	No symptoms (24 months)	Symptoms (24 months)	Total	*P* value
	(N = 46)	(N = 75)	(N = 121)	
Duration (days) from COVID-19 diagnosis to 24 months following COVID-19 infection, median [IQR]	749.0 [742.0–755.0]	746.0 [739.0–755.0]	749.0 [739.0–755.0]	0.327
Days from COVID-19-related symptom onset to diagnosis, median [IQR]	2.0 [0.0–4.0]	2.0 [0.0–5.0]	2.0 [0.0–4.0]	0.568
Sex				0.025
Male	25 (54.3%)	24 (32.0%)	49 (40.5%)	
Female	21 (45.7%)	51 (68.0%)	72 (59.5%)	
Age (years), median [IQR]	48.5 [36.0–58.0]	54.0 [39.5–62.0]	52.0 [39.0–61.0]	0.069
Age distribution (years)				0.416
20–29	7 (15.2%)	9 (12.0%)	16 (13.2%)	
30–39	9 (19.6%)	10 (13.3%)	19 (15.7%)	
40–49	8 (17.4%)	10 (13.3%)	18 (14.9%)	
50–59	13 (28.3%)	19 (25.3%)	32 (26.4%)	
60–70	9 (19.6%)	27 (36.0%)	36 (29.8%)	
Height (cm), median [IQR]	167.0 [158.0–175.0]	161.0 [156.5–167.0]	163.0 [157.0–170.5]	0.031
Weight (kg), median [IQR]	68.0 [55.0–75.0]	61.0 [54.5–69.0]	63.0 [55.0–72.5]	0.077
BMI (kg/m^2^), median [IQR]	24.0 [22.2–26.4]	23.9 [21.6–25.4]	24.0 [21.8–25.8]	0.402
Current smoker				1.000
Yes	1 (2.2%)	1 (1.3%)	2 (1.7%)	
No	45 (97.8%)	74 (98.7%)	119 (98.3%)	
Disease severity category				0.167
Asymptomatic	4 (8.7%)	1 (1.3%)	5 (4.1%)	
Mild	24 (52.2%)	38 (50.7%)	62 (51.2%)	
Moderate	15 (32.6%)	24 (32.0%)	39 (32.2%)	
Severe	3 (6.5%)	9 (12.0%)	12 (9.9%)	
Critical	0 (0.0%)	3 (4.0%)	3 (2.5%)	
Overall disease severity				0.211
≥ Severe	3 (6.5%)	12 (16%)	15 (12.4%)	
< Severe	43 (93.5%)	63 (84%)	106 (87.6%)	
Quarantine site during acute COVID-19 infection				0.084
Secondary or tertiary hospital	34 (73.9%)	64 (85.3%)	98 (81.0%)	
Therapeutic living center	12 (26.1%)	9 (12.0%)	21 (17.4%)	
Self-quarantine at home	0 (0.0%)	2 (2.7%)	2 (1.7%)	
COVID-19 vaccination history^a^				1.000
Yes	43 (93.5%)	70 (93.3%)	113 (93.4%)	
No	3 (6.5%)	5 (6.7%)	8 (6.6%)	
Number of COVID-19 vaccinations				0.846
0	3 (6.5%)	5 (6.7%)	8 (6.6%)	
2	12 (26.1%)	25 (33.3%)	37 (30.6%)	
3	30 (65.2%)	44 (58.7%)	74 (61.2%)	
4	1 (2.2%)	1 (1.3%)	2 (1.7%)	
Diagnosed with new disease since COVID-19 infection				0.969
Yes	4 (8.7%)	8 (10.7%)	12 (9.9%)	
No	42 (91.3%)	67 (89.3%)	109 (90.1%)	
Readmitted to hospital or health facility after the first acute COVID-19 illness				> 0.999
Yes	0 (0.0%)	0 (0.0%)	0 (0.0%)	
No	46 (100.0%)	75 (100.0%)	121 (100.0%)	
Re-infection from COVID-19				0.132
Yes	4 (8.7%)	1 (1.3%)	5 (4.1%)	
No	42 (91.3%)	74 (98.7%)	116 (95.9%)	

IQR, interquartile range; COVID-19, coronavirus disease 2019; BMI, body mass index.

^a^All individuals received vaccination after acute COVID-19 infection. The COVID-19 vaccines used were Astrazeneca (AZ), Moderna COVID-19 (mRNA-1273), and Pfizer BioNTech (BNT162b2).

### Characteristics of long COVID

**Figure 1:**
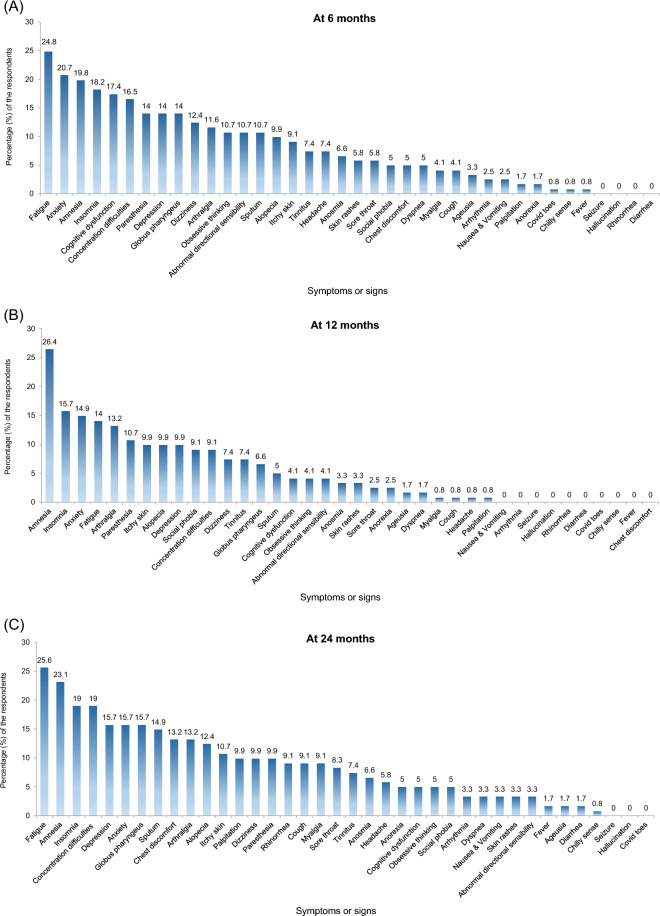
Distribution of patients with long COVID after (**A**) 6 months, (**B**) 12 months, and (**C**) 24 months following acute COVID-19 infection. COVID-19, coronavirus disease 2019.

**Figure 2:**
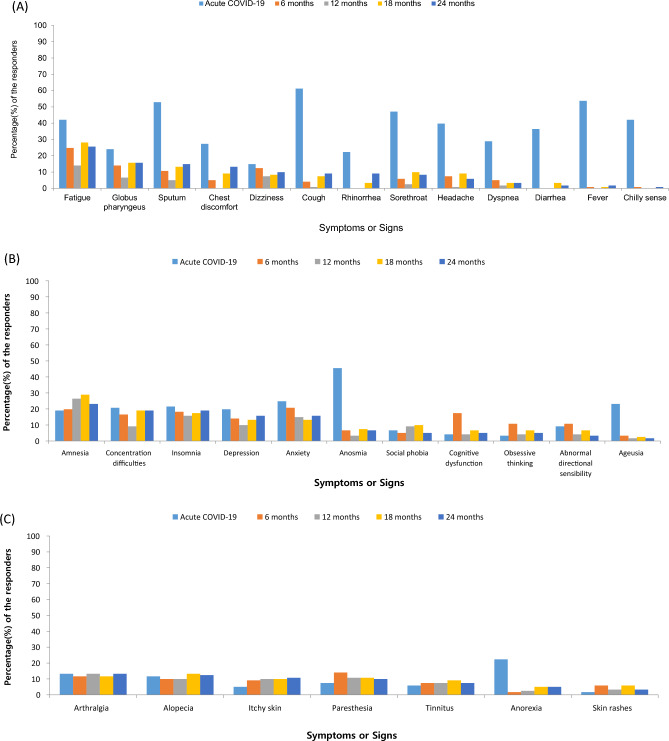
Symptoms identified during and after acute COVID-19 infection. (**A**) Distribution of constitutional symptoms. (**B**) Distribution of neuropsychiatric symptoms. (**C**) Distribution of other symptoms. COVID-19, coronavirus disease 2019.

Six months after acute COVID-19 infection, 72 patients (58.7%) had at least one long COVID symptom (Fig. 1A). After 12 months (Fig. 1B) and 24 months (Fig. 1C), 62 (51.2%) and 75 (62.0%) patients reported at least one symptom, respectively. The most common symptoms at 24 months were fatigue (31 patients; 25.6%), amnesia (28 patients; 23.1%), concentration difficulties (23 patients; 19.0%), and insomnia (23 patients; 19.0%). Additionally, 15 patients (12.4%) reported alopecia, 12 (9.9%) reported dizziness, and 12 (9.9%) reported paresthesia. Anosmia and ageusia were identified in eight (6.6%) and two (1.7%) patients, respectively (Fig. 1C). While cough, fever, sputum production, anosmia, and ageusia improved over time (Fig. 2A), patients reported a higher incidence of amnesia and concentration difficulties at the latest timepoint than they had earlier (Fig. 2B). The frequencies of arthralgia, alopecia, and paresthesia did not change significantly over the course of the study (Fig. 2C). Changes in symptoms recorded over time using a Sankey flow diagram at 6, 12, and 24 months following acute COVID-19 infection revealed that neuropsychological symptoms overlapped and persisted interacting with other long COVID symptoms depending on the time change (Supplementary Fig. S1).

### Impact of COVID-19 vaccination on long COVID symptom persistence

Out of 121 patients, 113 were confirmed to be fully vaccinated against COVID-19 by the 24-month timepoint following COVID-19 infection (eight patients were never vaccinated). All vaccinated patients received at least two doses. At 24 months, comparison between vaccinated and unvaccinated patients revealed no significant difference in major neuropsychiatric symptoms of long COVID (Table 2). For vaccinated patients, there was no significant difference in the frequency of major neuropsychiatric symptoms between patients who received fewer than three vaccine doses and those who received three or more (Table 3). Repeating this analysis with the unvaccinated patients included, there was still no significant difference in neuropsychiatric symptom frequency between patients who had received fewer or more than three vaccine doses (Supplementary Table S3).

**Table 2:** Impact of COVID-19 vaccination on long COVID symptoms.

Long COVID symptoms	Not vaccinated	Vaccinated	Total	*P* value
	(N = 8)	(N = 113)	(N = 121)	
Fatigue				1.000
Yes	2 (25.0%)	29 (25.7%)	31 (25.6%)	
No	6 (75.0%)	84 (74.3%)	90 (74.4%)	
Amnesia				0.786
Yes	0 (0.0%)	28 (24.8%)	28 (23.1%)	
No	8 (100.0%)	85 (75.2%)	93 (76.9%)	
Insomnia				0.341
Yes	0 (0.0%)	23 (20.4%)	23 (19.0%)	
No	8 (100.0%)	90 (79.6%)	98 (81.0%)	
Difficulty concentrating				0.341
Yes	0 (0.0%)	23 (20.4%)	23 (19.0%)	
No	8 (100.0%)	90 (79.6%)	98 (81.0%)	
Depression				1.000
Yes	1 (12.5%)	18 (15.9%)	19 (15.7%)	
No	7 (87.5%)	95 (84.1%)	102 (84.3%)	
Anxiety				0.447
Yes	0 (0.0%)	19 (16.8%)	19 (15.7%)	
No	8 (100.0%)	94 (83.2%)	102 (84.3%)	
Cognitive dysfunction				1.000
Yes	0 (0.0%)	6 (5.3%)	6 (5.0%)	
No	8 (100.0%)	107 (94.7%)	115 (95.0%)	
Anosmia				1.000
Yes	1 (12.5%)	7 (6.2%)	8 (6.6%)	
No	7 (87.5%)	106 (93.8%)	113 (93.4%)	
Ageusia				0.291
Yes	1 (12.5%)	1 (0.9%)	2 (1.7%)	
No	7 (87.5%)	112 (99.1%)	119 (98.3%)	

COVID-19, coronavirus disease 2019.

Data are presented as n (%).

**Table 3:** Long COVID symptoms in 113 COVID-19-vaccinated patients stratified by number of vaccination doses.

Long COVID symptoms	< 3 vaccination doses	≥ 3 vaccination doses	Total	*P* value
	(N = 36)	(N = 77)	(N = 113)	
Fatigue				1.000
Yes	9 (25.0%)	20 (26.0%)	29 (25.7%)	
No	27 (75.0%)	57 (74.0%)	84 (74.3%)	
Amnesia				0.786
Yes	10 (27.8%)	18 (23.4%)	28 (24.8%)	
No	26 (72.2%)	59 (76.6%)	85 (75.2%)	
Insomnia				0.678
Yes	6 (16.7%)	17 (22.1%)	23 (20.4%)	
No	30 (83.3%)	60 (77.9%)	90 (79.6%)	
Difficulty concentrating				0.276
Yes	10 (27.8%)	13 (16.9%)	23 (20.4%)	
No	26 (72.2%)	64 (83.1%)	90 (79.6%)	
Depression				1.000
Yes	6 (16.7%)	12 (15.6%)	18 (15.9%)	
No	30 (83.3%)	65 (84.4%)	95 (84.1%)	
Anxiety				1.000
Yes	6 (16.7%)	13 (16.9%)	19 (16.8%)	
No	30 (83.3%)	64 (83.1%)	94 (83.2%)	
Cognitive dysfunction				1.000
Yes	2 (5.6%)	4 (5.2%)	6 (5.3%)	
No	34 (94.4%)	73 (94.8%)	107 (94.7%)	
Anosmia				0.541
Yes	1 (2.8%)	6 (7.8%)	7 (6.2%)	
No	35 (97.2%)	71 (92.2%)	106 (93.8%)	
Ageusia				1.000
Yes	0 (0.0%)	1 (1.3%)	1 (0.9%)	
No	36 (100.0%)	76 (98.7%)	112 (99.1%)	

### Psychiatric status assessment

**Figure 3:**
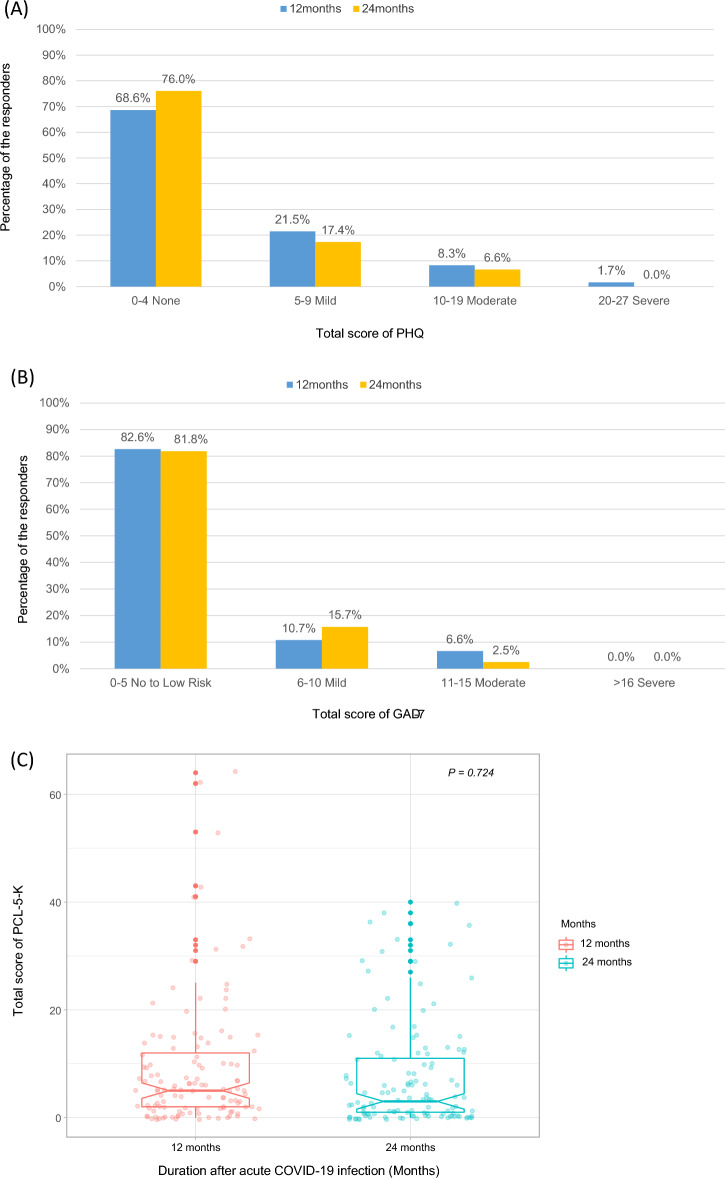
Distribution of total (**A**) PHQ-9, (**B**) GAD-7, and (**C**) PCL-5-K scores (N = 121). PHQ-9, Korean version of the Patient Health Questionnaire-9 (depression screening for individuals at risk); GAD-7, Generalized Anxiety Disorder-7; PCL-5-K, Korean version of Post-Traumatic Stress Disorder Checklist-5.

At 12 months following initial COVID-19 infection, 12 out of 121 patients (10.0%) reported moderate to severe depression; this number had decreased to 8 patients (6.6%) by 24 months (Fig. 3A). Patients with persistent long COVID symptoms at 24 months had higher PHQ-9 scores than those without (*P* < 0.001; Supplementary Table S4). The PHQ-9 scores of participants without severe initial illness did not change significantly over time. However, higher PHQ-9 scores were initially observed in the severe illness group; these improved to the levels of the group with milder illness by 12 month follow-up (Supplementary Fig. S2).

At 12 months after infection, 6.6% of patients had moderate or higher GAD-7 scores, but this proportion was reduced to 3.3% at 24 months (Fig. 3B). Patients with long COVID symptoms at 24 months had higher GAD-7 scores than those without (*P* < 0.001; Supplementary Table S4). There was no difference in the GAD-7 score over time in the patient group with less-than-severe disease. Similar to PHQ-9 findings, the GAD-7 score of the severe illness group was initially elevated but improved to a similar level as the milder disease groups over time (Supplementary Fig. S2).

The median PCL-K-5 score at 12 months was 5 (IQR 2.0–12.0), while at 24 months, it was 3 (1.0–11.0), indicating a tendency to improve with time (*P* = 0.724; Fig. 3C). Both disease severity groups improved overall with time following acute COVID-19 infection (Supplementary Fig. S2).

### Quality of life and lifestyle changes

Over time from 12 to 24 months after acute COVID-19 infection, five EQ5D categories revealed an improvement in quality of life (Supplementary Fig. S3). However, pain/discomfort and anxiety/depression categories accounted for 32.2% of the total patients who reported being affected by the quality of life 24 months after COVID-19 infection, and they had a more significant impact on the quality of life than mobility, self-care, and usual activities of EQ5D categories (Supplementary Fig. S4). Two years after COVID infection, reports of trouble walking or ascending steps and challenges in remembering or concentrating were significantly more common in the group that also reported other long COVID symptoms than in the group without persistent symptoms (*P* = 0.006 and *P* < 0.001, respectively; Supplementary Table S5). A higher percentage of patients in the long COVID group attempted to eat healthy food and exercise more frequently than those in the group that was symptom-free after 2 years, although this difference was not significant (*P* > 0.05; Supplementary Table S6).

## Discussion

In this prospective cohort study, we have confirmed the previous findings of others that COVID-19 diagnosis is associated with an increased risk of various neurological and psychiatric diagnoses in the 6–12 months following illness^27–29^ and showed that psychological symptoms may persist for more than 2 years after acute COVID-19 infection. Even after 24 months, a decline in quality of life may persist.

Previous systematic reviews have revealed that fatigue, dyspnea, difficulty concentrating, anxiety, sleep disorders, memory impairment, and cognitive impairment are the most common long COVID symptoms^30^. Fatigue and neuropsychological symptoms may become more prevalent than other symptoms^27,31^.

In our study, 51.2% of patients experienced only mild disease severity from COVID-19 infection and 93.4% were subsequently vaccinated, but 62.0% had at least one long COVID symptom that persisted until 24 months, with fatigue being the most prevalent. Similar results were seen in a previous study from China that also followed patients for 2 years after COVID-19 infection^32^.

Other studies have revealed multiple biological pathways that could explain the broad array of neurological disorders seen in the setting of long COVID, including disruptions in cognition, memory, and mental health^33^.

In our study, the most prevalent neuropsychiatric symptoms 24 months after acute COVID-19 infection were amnesia and difficulty concentrating. Previous research has shown that patients infected with the SARS-CoV-2 ancestral strain developed increased incidence of cognitive impairment compared with that of uninfected study participants^34^. As SARS-CoV-2 infection has the potential to increase and contribute to dementia and “brain fog,” including concentration difficulty and cognitive impairment^35,36^, more long-term follow-up studies are needed. More effective interventions will be necessary to reduce the burden of long COVID, particularly that arising from neurodegenerative and neuropsychological diseases.

Many symptoms of long COVID may be interrelated. For example, this study showed a similar pattern of change over time for concentration difficulties, depression, and insomnia. Thus, patients could be experiencing difficulty concentrating that is directly caused or exacerbated by depression and insomnia.

Our findings showed the characteristics of original strain associated long COVID, and reveal that neuropsychological symptoms can be long-term problem even 24 months following acute COVID-19 infection. Although the prevalence of long COVID was reported to be less frequent after infection with the now-prevalent Omicron strain than with Delta strain infection^37^, the risk of persistence of psychotic disorders, cognitive impairments, and dementia remains high two years after SARS-CoV-2 infection, and neurological and psychiatric outcomes are similar during delta and omicron waves^38^. Not only in the original variant strain infected younger patients^39^, but memory loss can be a continuous problem in the younger age group infected with omicron variant^40^. Even with less severe variants COVID-19 infection or COVID-19 infection in younger age group, neuropsychological symptoms may persist in the long term and can become a problem affecting to social and healthcare consequences. Therefore, continuous long-term follow-up studies associated with different variants are needed to identify the impact of SARS-CoV-2 infection.

A previous study showed no significant differences in long COVID symptoms, including memory loss, brain fog, and difficulty concentrating, between hospitalized and nonhospitalized patients 2 years after acute infection^41^. We also observed no significant difference in the symptoms between two groups based on illness severity. This indicates that long COVID could become a social burden in the long run, regardless of disease severity during the acute infection state, especially in patients with mild disease severity, which accounts for the majority of COVID-19-infected patients.

Long COVID is known to be associated with a decline in quality of life^14,42^. In particular, neuropsychiatric symptoms have a significantly negative impact on health-related quality of life (HRQoL)^29,43^. One study reported that at 2 years, survivors with long COVID had lower HRQoL, more mental health abnormalities, and increased health care usage after discharge than survivors without long COVID. COVID-19 survivors still experienced more symptoms and problems with pain or discomfort, as well as anxiety or depression, than those who had not been infected with COVID-19. However, HRQoL improved over time, with the proportion of participants reporting symptoms of anxiety or depression declining significantly from 23% at 6 months to 12% at 2 years^32^.

Consistent with the previous study^32^, our findings indicate that the impact of COVID-19 on quality of life improves over time. However, we found that at 24 months, the proportion of patients with anxiety, depression, pain, or discomfort was still higher than in other domains. In our cohort, 32.2% of patients still reported anxiety or depression affecting their quality of life 24 months after acute illness.

Results from a UK survey suggest that vaccination following infection may reduce the symptom burden of long COVID after the first dose, with sustained improvement after a second dose^20^. Individuals who were not vaccinated before infection experienced long COVID symptoms for up to 2 years, with evidence of increased symptom risk compared with vaccinated controls^44^. People with long COVID may experience dysregulation of the immune system and therefore benefit from autoimmune processes being “reset” by vaccination (although whether this is long-lasting remains to be established). The antibody response may also eliminate any leftover viral reservoir^45^. A previous systematic review included patients with a follow-up period of 18 months after acute COVID-19 infection and demonstrated a possible benefit of vaccination on long COVID, particularly in terms of confusion and difficulty concentrating^16^. In our study, 93.4% of all respondents received at least two doses of vaccine after acute COVID-19 infection, 62% of patients complained of more than one suspected long COVID symptom, and neuropsychiatric symptoms could linger for at least 24 months following acute COVID-19 infection. Our analysis found no difference in the incidence of long COVID based on vaccination status or the number of vaccine doses, supporting a previous systematic review that found the impact of vaccination in people with pre-existing long COVID symptoms to be negligible^46^. However, due to our small number of unvaccinated patients, a study with a higher proportion of unvaccinated patients will be required to determine the effectiveness of vaccination on long COVID. Future studies on COVID-19 vaccination could help prevent neuropsychiatric symptoms and improve the quality of life for long COVID patients.

Our data should be interpreted considering the study’s limitations. First, it is a single-center study with participants recruited during the early phases of the global pandemic, and therefore the findings may not directly apply to the long-term health outcomes of individuals infected with different SARS-CoV-2 variants. Second, patients with long COVID may have participated more actively in the survey, potentially leading to higher reported incidence rates of long COVID in this cohort. Third, the lack of uninfected and unvaccinated controls restricts the capacity to evaluate a direct link between SARS-CoV-2 infection and overall and particular post–COVID-19 symptoms 24 months later, as well as the effect of COVID-19 vaccination on patients with long COVID. Therefore, future research should aim to include an uninfected control group, as well as a larger number of unvaccinated patients.

Despite these limitations, the study’s merit lies in its well-defined, long-term follow-up design involving participants of diverse ages and varying disease severity during acute COVID-19 infection. Our research is significant as a long-term follow-up study beginning in the early days of the COVID-19 pandemic. Additionally, the analysis included confirmed vaccination and reinfection history from documented data. Long-term follow-up of patients with long COVID will be necessary to understand its pathophysiological mechanisms, with the eventual goal of developing treatments that can help alleviate long COVID symptoms.

## Conclusions

Our study shows that even 24 months after COVID-19 infection, neuropsychological symptoms, in particular, can persist more frequently than other symptoms, and there may still be a decrease in quality of life among patients who have received COVID-19 vaccination. Further large-scale long-term cohort studies including uninfected or unvaccinated controls are needed to understand the long-term impact of COVID-19.

### Supplementary Information

Supplementary Figures.

Supplementary Tables.

## Data Availability

The datasets generated during and/or analyzed during the current study are available from the corresponding author upon reasonable request.
