# Supplementary material for: Characteristics of long COVID and the impact of COVID-19 vaccination on long COVID 2 years following COVID-19 infection: prospective cohort study
Source: Sci Rep. 2024 Jan 9;14:854. doi: 10.1038/s41598-023-50024-4 (PMC10774352; doi:10.1038/s41598-023-50024-4)
Supplement: Supplementary file 1 — Supplementary Figures. [file 41598_2023_50024_MOESM1_ESM.docx]

Supplementary Fig. S1. Overview of Sankey flow diagrams: Focusing on the 9 major clinical characteristics of long COVID at 6, 12, and 24 months after acute COVID-19 infection in 121 cohort patients. Sankey diagrams show the changes of each long COVID symptom distribution and the interaction of each long COVID symptom depending on the change in time.

**
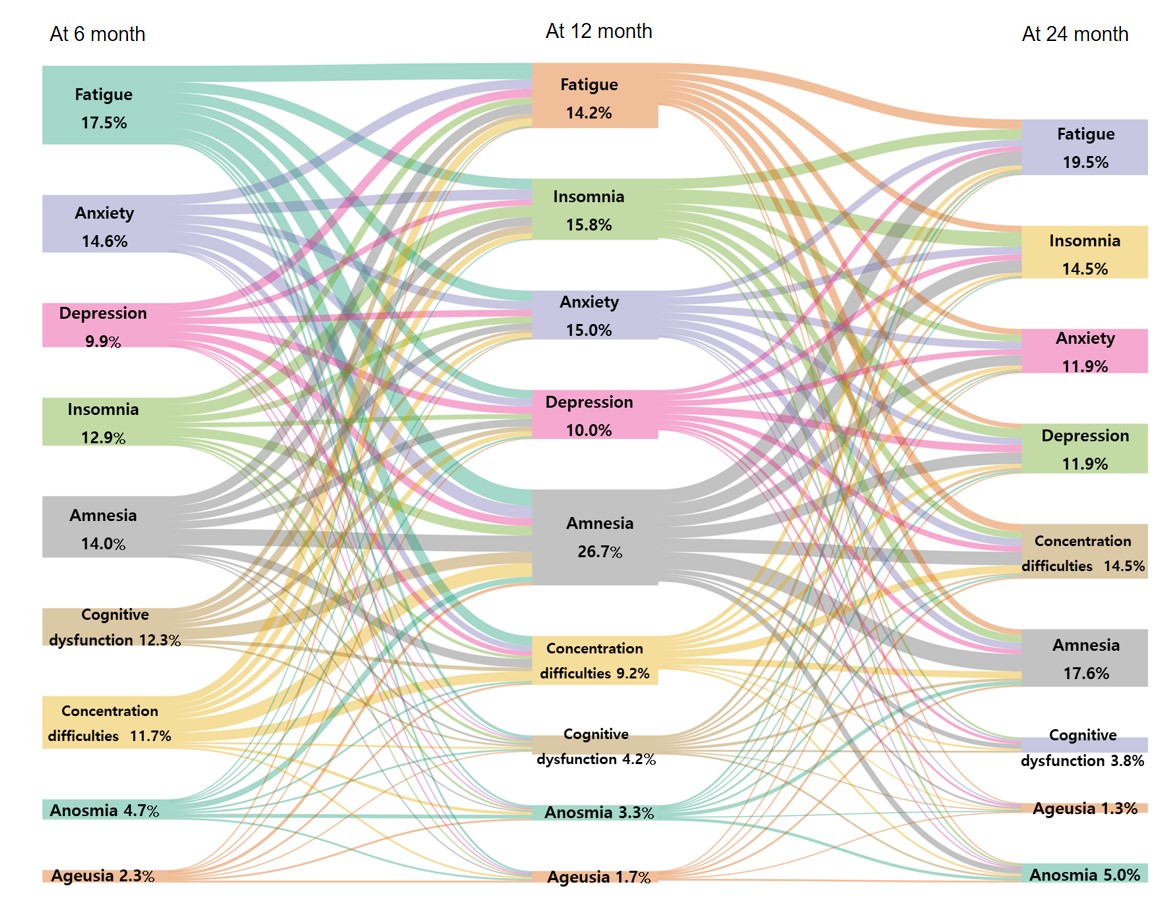
**

Supplementary Fig. S2. Total (A) PHQ, (B) GAD-7, and (C) PCL-5-K scores at 12, 18, and 24 months after acute COVID-19 infection according to disease severity < severe and disease severity ≥ severe. Violin-plot show the distribution of each individual’s scores and peak score point.

(A)
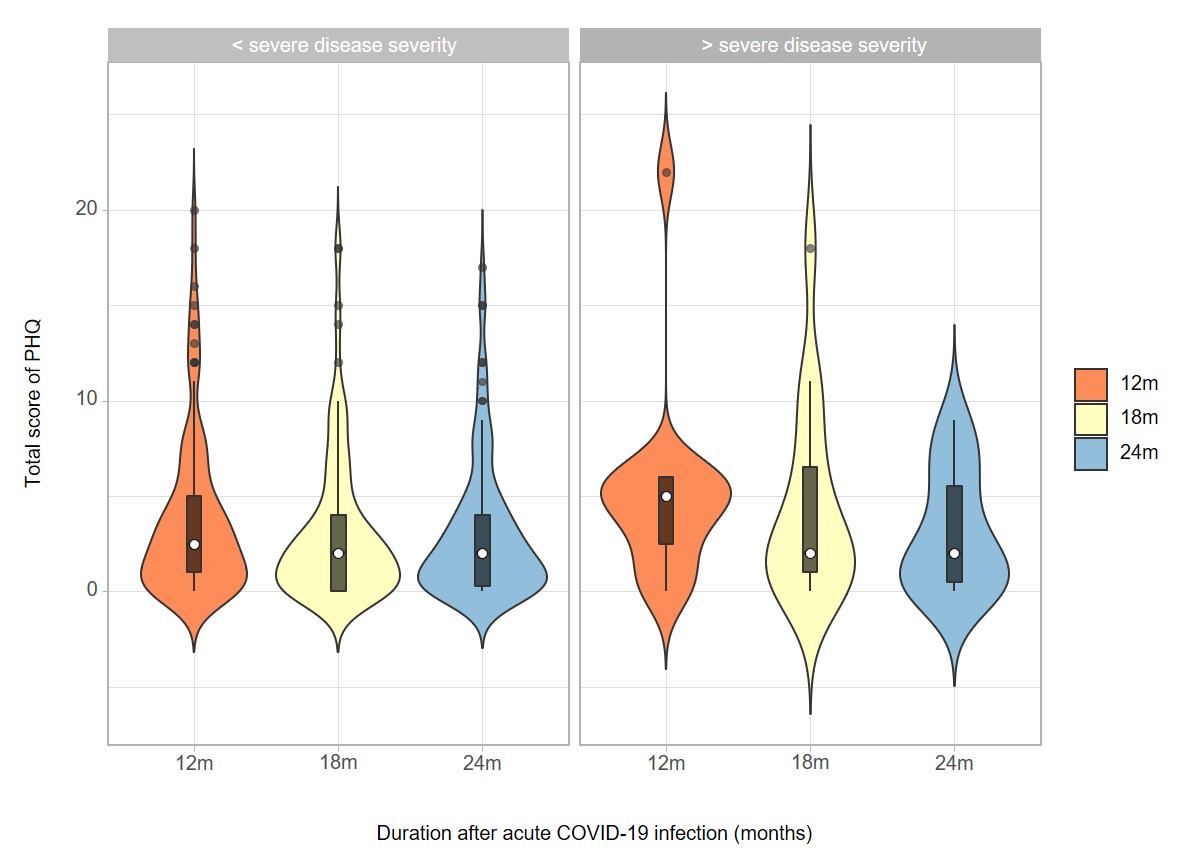


(B)


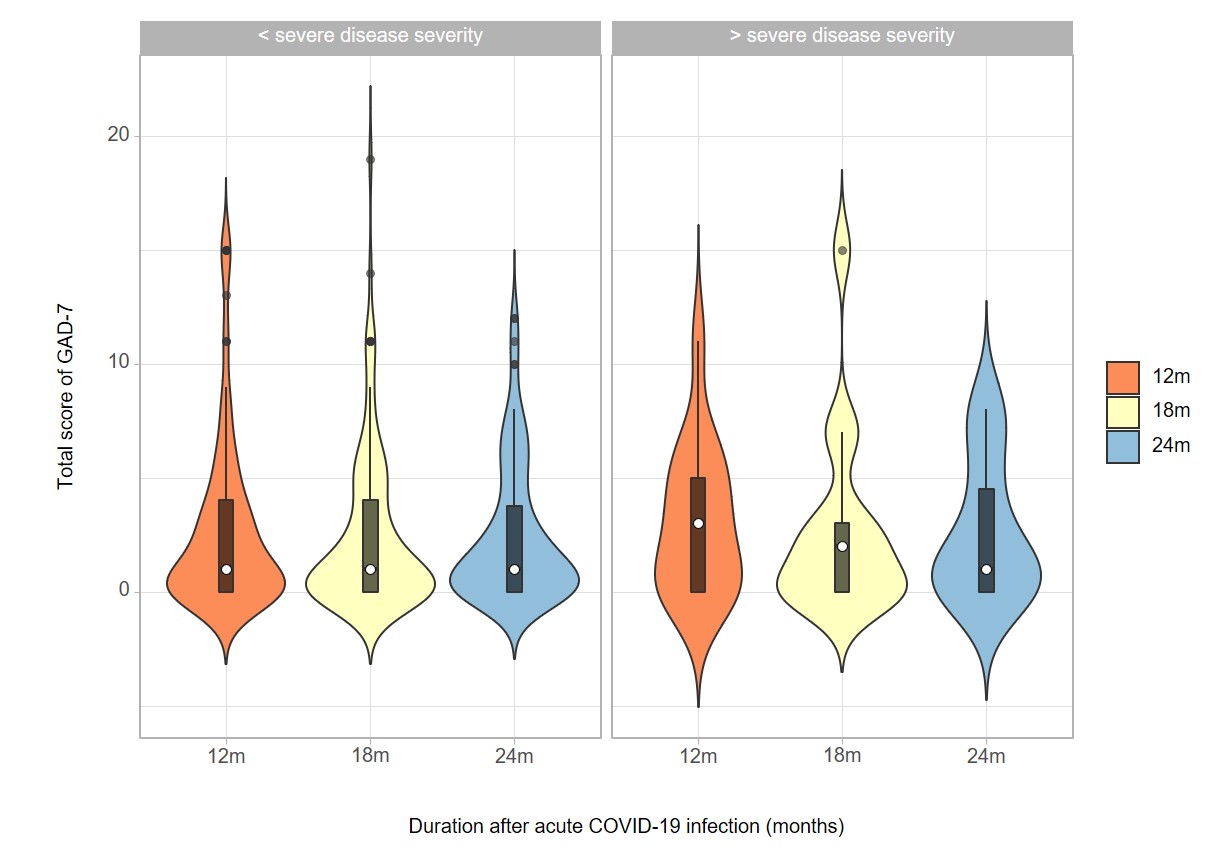


(C)


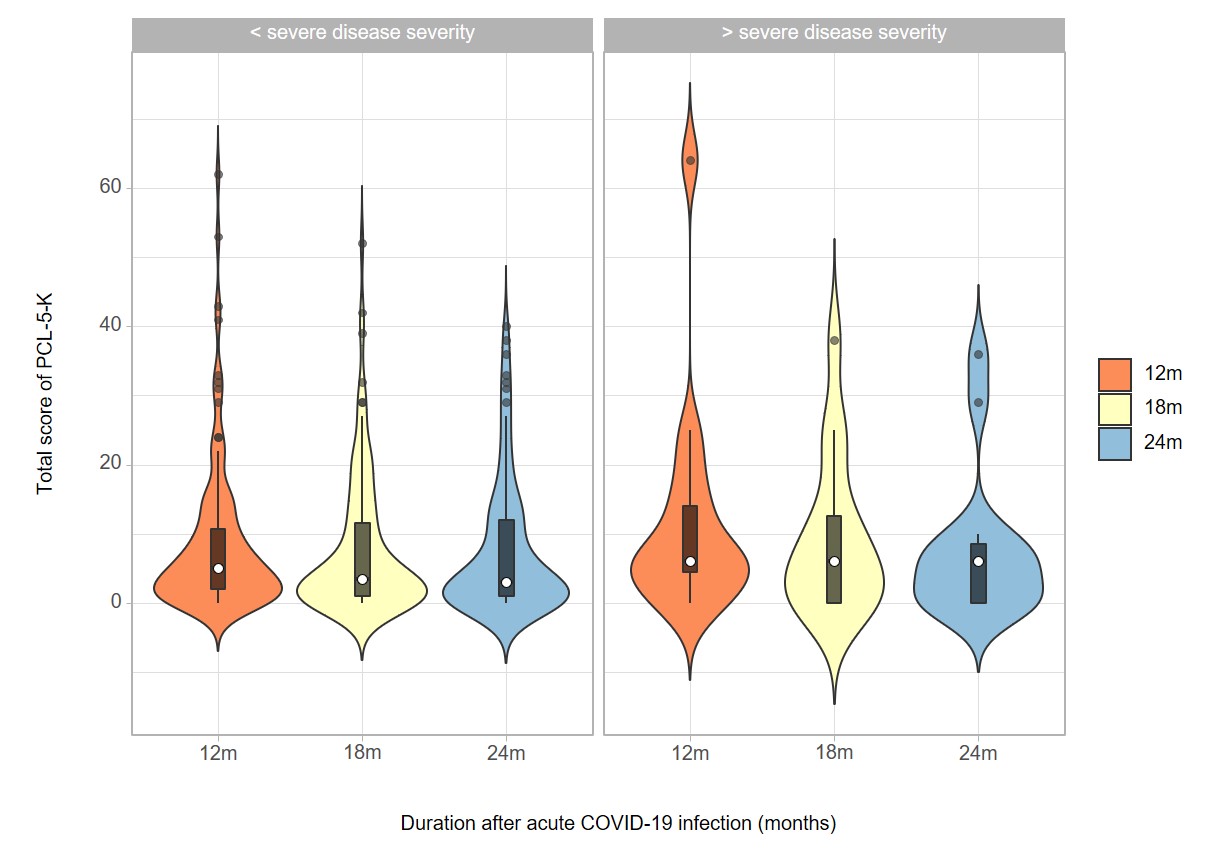


Supplementary Fig. S3. Assessment of quality of life (EQ-5D) in a total of 121 cohort patients according to (A) mobility, (B) self-care, (C) usual activities, (D) pain/discomfort, and (E) anxiety/depression after 12 and 24 months of acute COVID-19 infection.

(A)


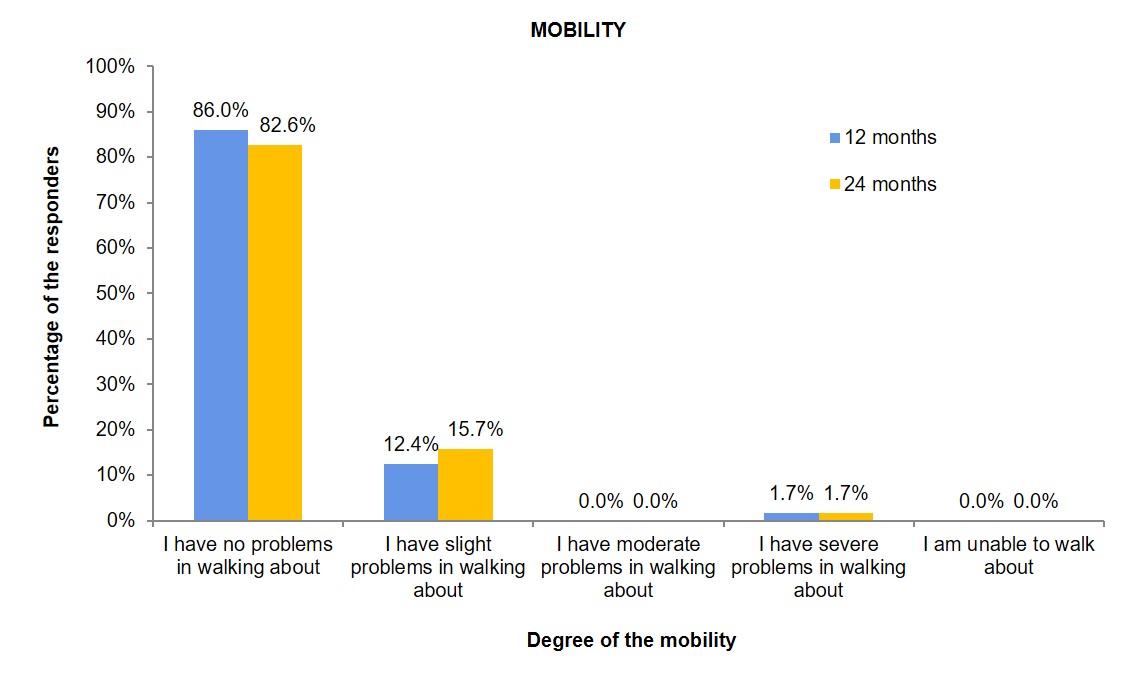


(B)


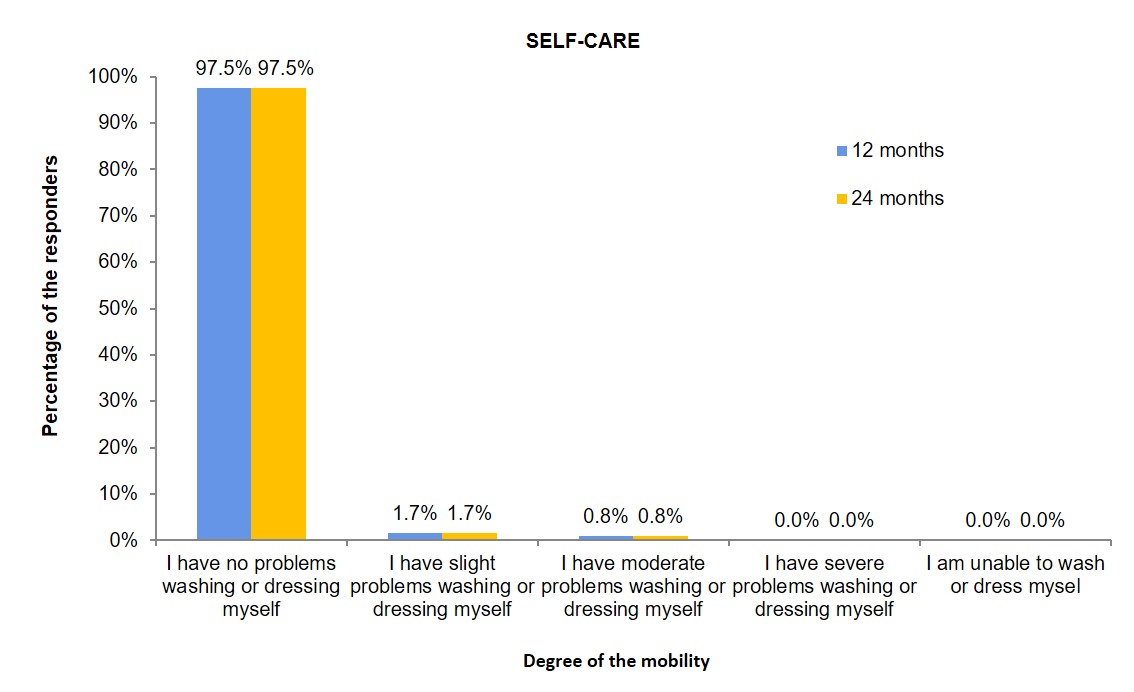


(C)


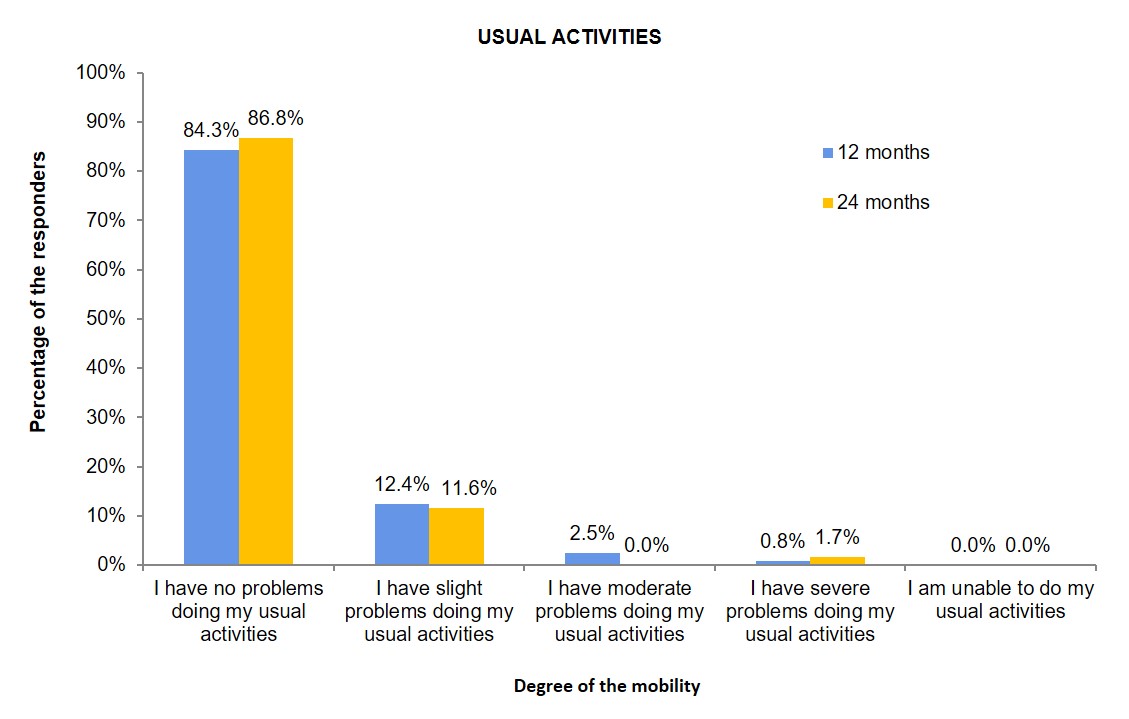


(D)


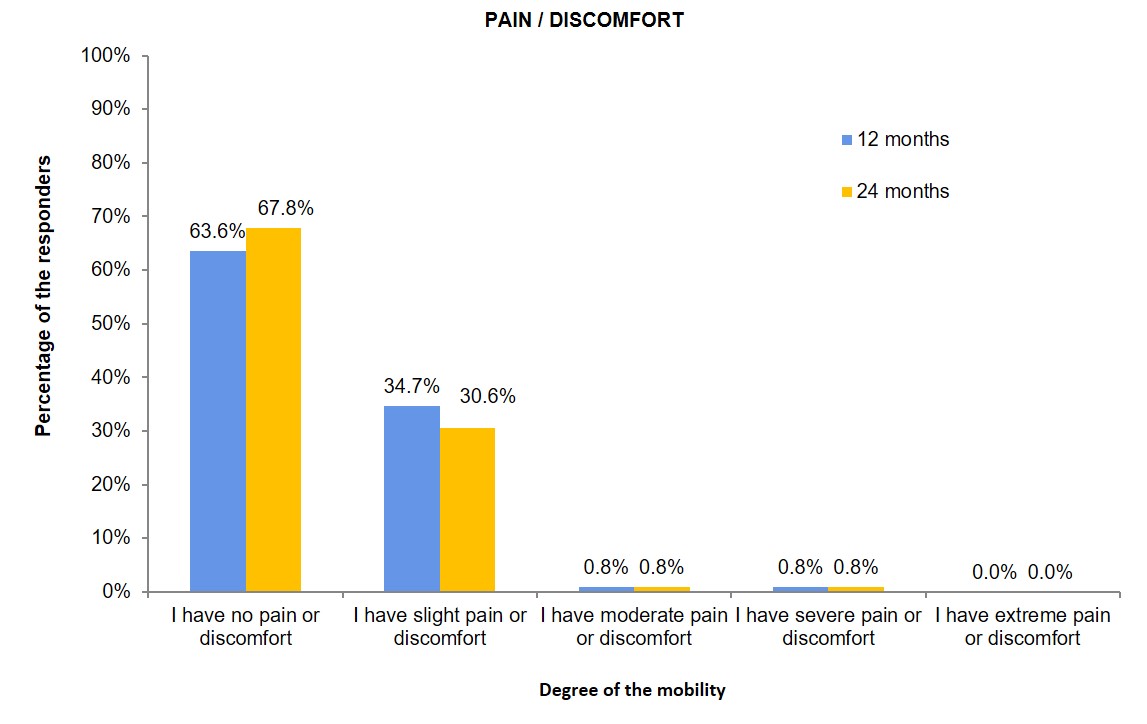


(E)

**
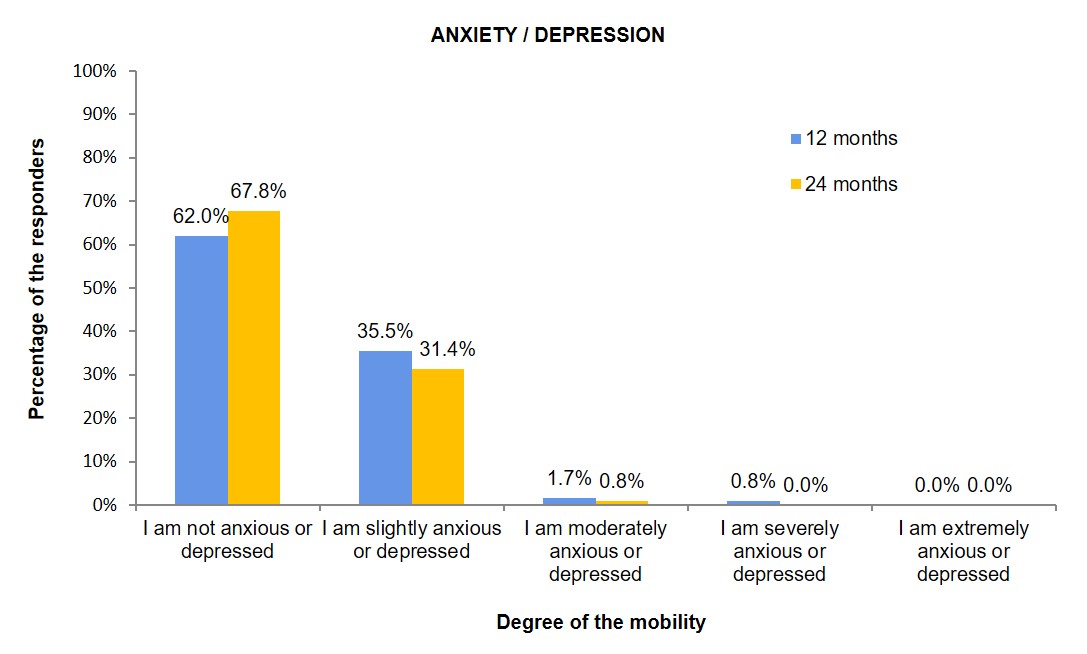
**

Supplementary Fig. 4. Distribution of EQ-5D-5L (A) median values and (B) degree of symptoms at 24 months after acute COVID-19 infection in all 121 patients.

(A)

**
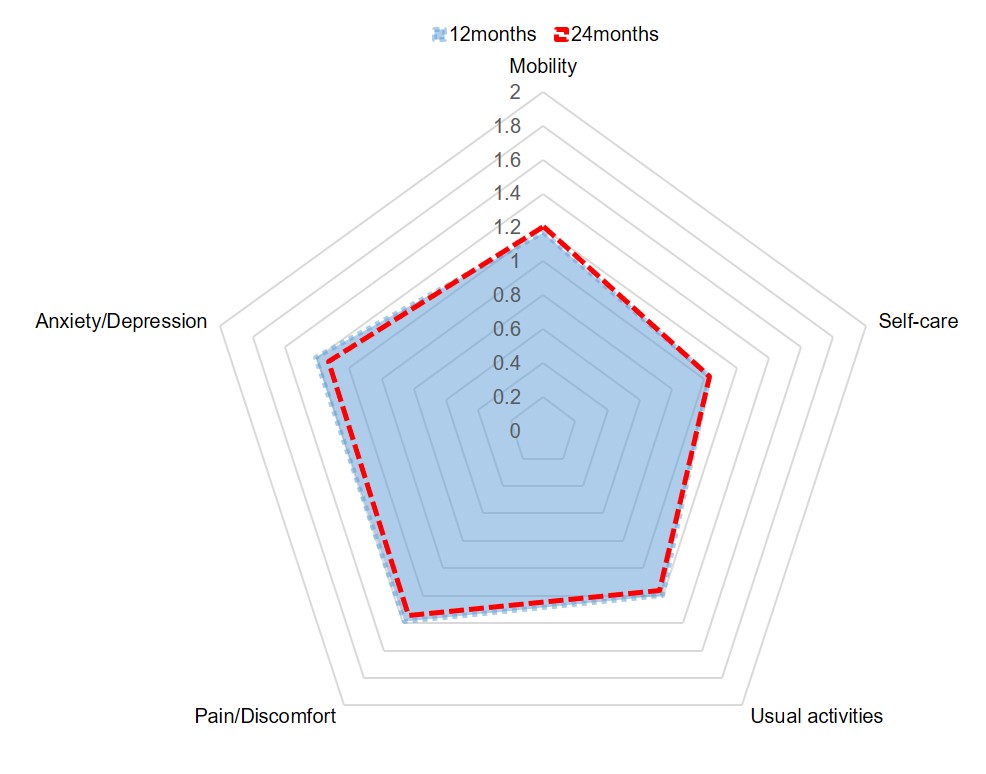
**

(B)

**
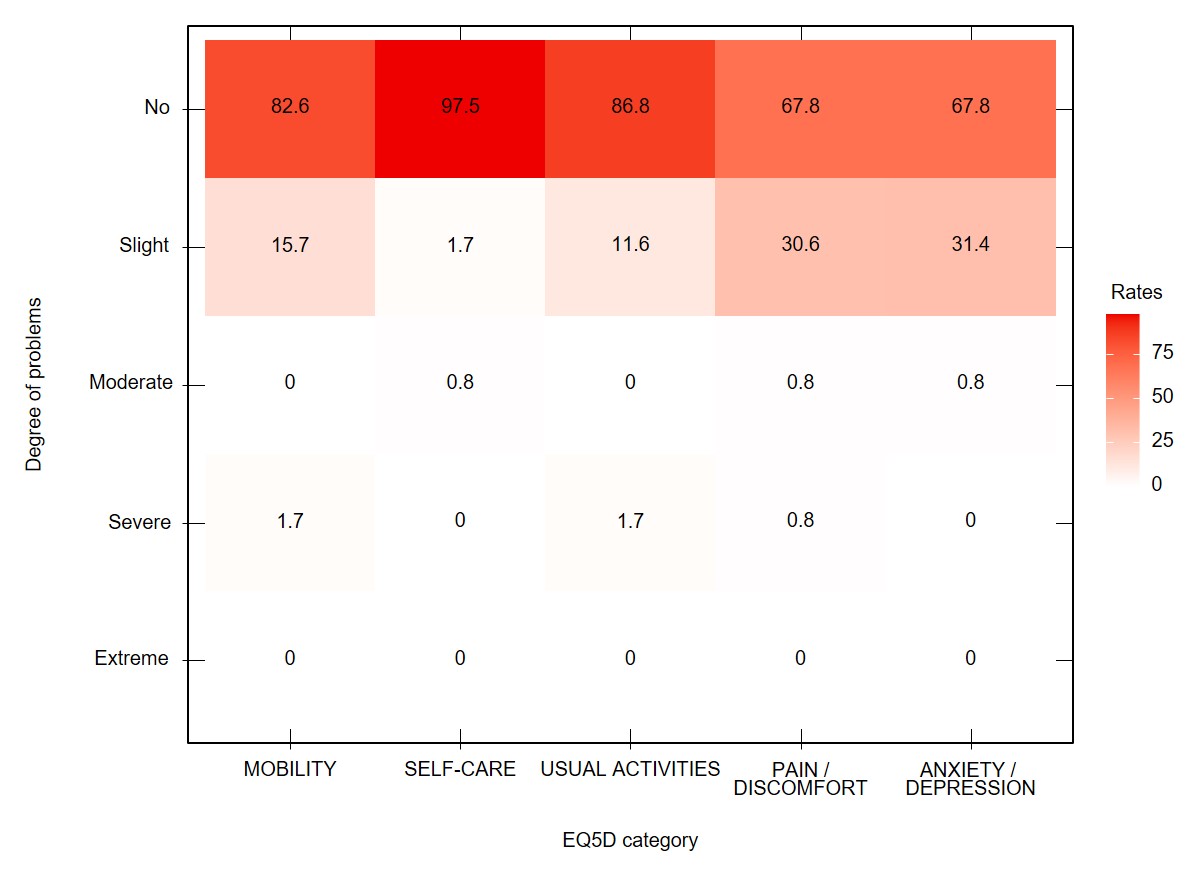
**
