# Supplementary material for: Characteristics of long COVID and the impact of COVID-19 vaccination on long COVID 2 years following COVID-19 infection: prospective cohort study
Source: Sci Rep. 2024 Jan 9;14:854. doi: 10.1038/s41598-023-50024-4 (PMC10774352; doi:10.1038/s41598-023-50024-4)
Supplement: Supplementary file 2 — Supplementary Tables. [file 41598_2023_50024_MOESM2_ESM.docx]

Supplementary Table S1: Survey form administered to participants.

| 1. Participant’s information, participant identification # |
| --- |
| Name:  Male □ Female □  Age:­­___years old  Height/weight:__cm/ __kg  Smoking: □ Yes □ No  Quarantine site during acute COVID-19 infection:  □ At home □ Therapeutic living center □ Secondary or tertiary hospital |
| 2. About you and your COVID-19 illness |
| Survey completion date (DD/MM/YYYY):  What is your date of birth: |
| Have you been vaccinated against COVID-19? □ Yes □ No □ Not sure  If yes, how many times have you had the COVID-19 vaccine? [number]  Estimated date of the last vaccine dose received: DD/MM/20YY  Which type of COVID-19 vaccine did you receive: □ AstraZeneca □ Pfizer-BioNTech □ Janssen □ Moderna □ Other (name): ________ □ Not sure |
| Have you been vaccinated against influenza within last 6 months? □ Yes □ No □ Not sure |
| Roughly, on what day did you first experience symptoms of COVID-19? DD/MM/20YY  Were you admitted to the hospital due to COVID-19 or diagnosed with COVID-19 during a hospital admission? □ Yes □ No  • Roughly, on what day were you first admitted to the hospital? DD/MM/20YY  • Roughly, on what day were you first discharged from the hospital? DD/MM/20YY  • Have you been readmitted to hospital or health facility after your first acute COVID-19 illness?  □ Yes □ No  If yes, how many times: [number]  If ever admitted to a hospital/health facility for COVID-19, were you admitted to intensive care (ICU/ITU)? □ Yes □ No □ Not sure  • Have you been diagnosed with any new disease since having COVID-19? □ Yes □ No  • Have you had a re-infection from COVID-19? □ Yes □ No |
| 3. About your current health |
| Do you feel fully recovered from COVID-19? □ Strongly disagree □ Disagree □ Neither disagree nor agree □ Agree □ Strongly Agree |
| 4. Since having COVID-19, have you been diagnosed with any of these? |
| Heart attack □ Yes □ No  Stroke or mini stroke/TIA □ Yes □ No  Kidney problems □ Yes □ No  Deep vein thrombosis (DVT, “Clot in leg”) □ Yes □ No  Pulmonary embolism (PE, “Clot in lung”) □ Yes □ No  Other condition? (please specify)__________________ |
| 5. Within the last 7 days, have you had any of these symptoms? (that you did not experience before onset of your COVID-19 illness) |
| Fever □ Yes □ No  Chills □ Yes □ No  Muscle pain □ Yes □ No  Joint pain or swelling □ Yes □ No  Fatigue □ Yes □ No  Cough □ Yes □ No  Sore throat □ Yes □ No  Rhinorrhea □ Yes □ No  Sputum □ Yes □ No  Dyspnea □ Yes □ No  Palpitations □ Yes □ No  Arrhythmia □ Yes □ No  Chest discomfort □ Yes □ No  Headache □ Yes □ No  Dizziness □ Yes □ No  Cognitive dysfunction □ Yes □ No  Difficulty concentrating □ Yes □ No  Amnesia □ Yes □ No  Abnormal directional sensibility □ Yes □ No  Seizure □ Yes □ No  Paresthesia □ Yes □ No  Globus pharyngeus □ Yes □ No  Hallucination □ Yes □ No  Problems sleeping □ Yes □ No  Social phobia □ Yes □ No  Depression □ Yes □ No  Anxiety □ Yes □ No  Obsessive thinking □ Yes □ No  Anorexia □ Yes □ No  Diarrhea □ Yes □ No  Nausea or vomiting □ Yes □ No  Loss of smell □ Yes □ No  Loss of taste □ Yes □ No  Tinnitus □ Yes □ No  Hair loss □ Yes □ No  Skin rashes □ Yes □ No  Itchy skin □ Yes □ No  Swollen fingers or toes □ Yes □ No |
| 6. About your health  Under each heading, please tick the ONE box that best describes your health TODAY |
| MOBILITY  □ I had no problems in walking about.  □ I had slight problems in walking about.  □ I had moderate problems in walking about.  □ I had severe problems in walking about.  □ I was unable to walk about. |
| SELF-CARE  □ I had no problems washing or dressing myself.  □ I had slight problems washing or dressing myself.  □ I had moderate problems washing or dressing myself.  □ I had severe problems washing or dressing myself.  □ I was unable to wash or dress myself. |
| USUAL ACTIVITIES (e.g., work, study, housework, family, or leisure activities)  □ I had no problems doing my usual activities.  □ I had slight problems doing my usual activities.  □ I had moderate problems doing my usual activities.  □ I had severe problems doing my usual activities.  □ I was unable to do my usual activities. |
| PAIN/DISCOMFORT  □ I had no pain or discomfort.  □ I had slight pain or discomfort.  □ I had moderate pain or discomfort.  □ I had severe pain or discomfort.  □ I had extreme pain or discomfort. |
| ANXIETY/DEPRESSION  □ I was not anxious or depressed.  □ I was slightly anxious or depressed.  □ I was moderately anxious or depressed.  □ I was severely anxious or depressed.  □ I was extremely anxious or depressed. |
| • We would like to know how good or bad your health is TODAY.  • This scale is numbered from 0 to 100.  • 100 means the best health you can imagine. 0 means the worst health you can imagine.  • Mark an X on the scale to indicate how your health is TODAY.  • Now, please write the number you marked on the scale in the box below.  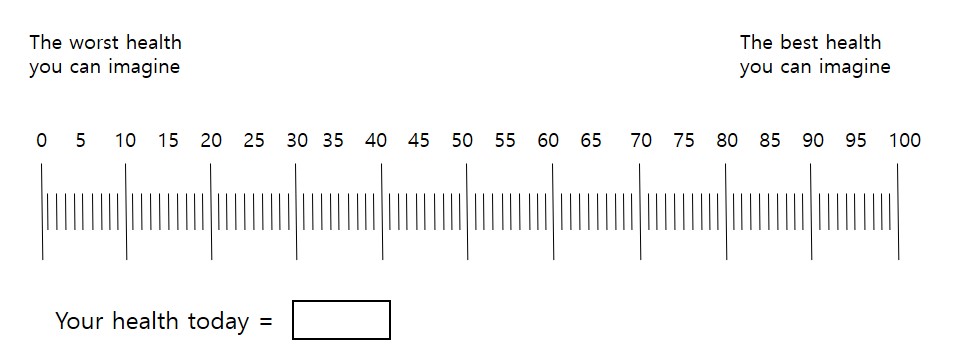 |
| 7. Breathlessness and tiredness  Please tick ONE box that best describes how breathless you feel today |
| Not troubled by breathlessness except on strenuous exercise □  Short of breath when hurrying or when walking up a slight hill □  Walking slower than most people of my age because of breathlessness or have to stop for breath when walking at my normal pace □  Stopping for breath after walking 100 yards (90–100 meters), or after a few minutes on level ground □  Too breathless to leave the house or breathless when dressing/undressing □ |
| Please rate the intensity of your fatigue on average over the last 24 h on a scale from 0 to 10.  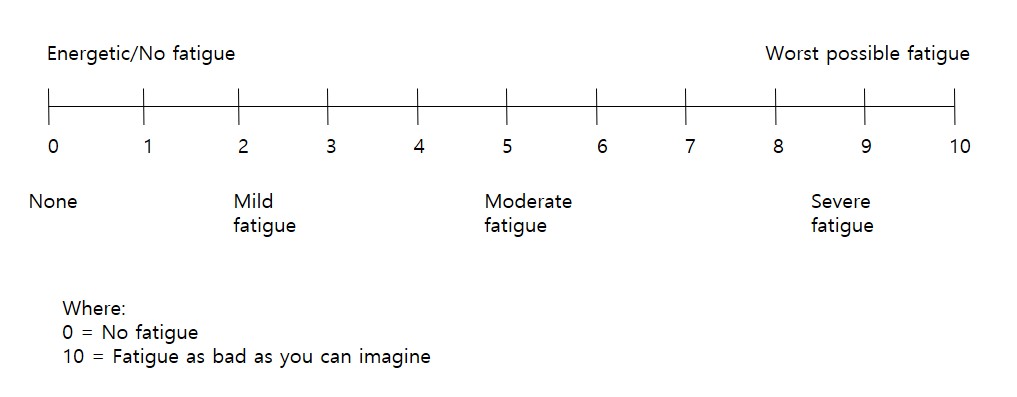 |
| 8. The next questions ask about difficulties you may have doing certain activities because of a HEALTH PROBLEM. (mark the correct answer with a tick in the box) |
| Do you have difficulty seeing even if wearing glasses?  □ No, no difficulty □ Yes, some difficulty □ Yes, a lot of difficulty □ Cannot do at all |
| Do you have difficulty hearing, even if using a hearing aid?  □ No, no difficulty □ Yes, some difficulty □ Yes, a lot of difficulty □ Cannot do at all |
| Do you have difficulty walking or climbing steps?  □ No, no difficulty □ Yes, some difficulty □ Yes, a lot of difficulty □ Cannot do at all |
| Do you have difficulty remembering or concentrating?  □ No, no difficulty □ Yes, some difficulty □ Yes, a lot of difficulty □ Cannot do at all |
| Do you have difficulty with self-care, such as washing all over or dressing?  □ No, no difficulty □ Yes, some difficulty □ Yes, a lot of difficulty □ Cannot do at all |
| Using your usual (customary) language, do you have difficulty communicating? For example, understanding or being understood?  □ No, no difficulty □ Yes, some difficulty □ Yes, a lot of difficulty □ Cannot do at all |
| 9. Have you made lifestyle changes since your COVID-19 infection? (mark the correct answer with a tick in the box)  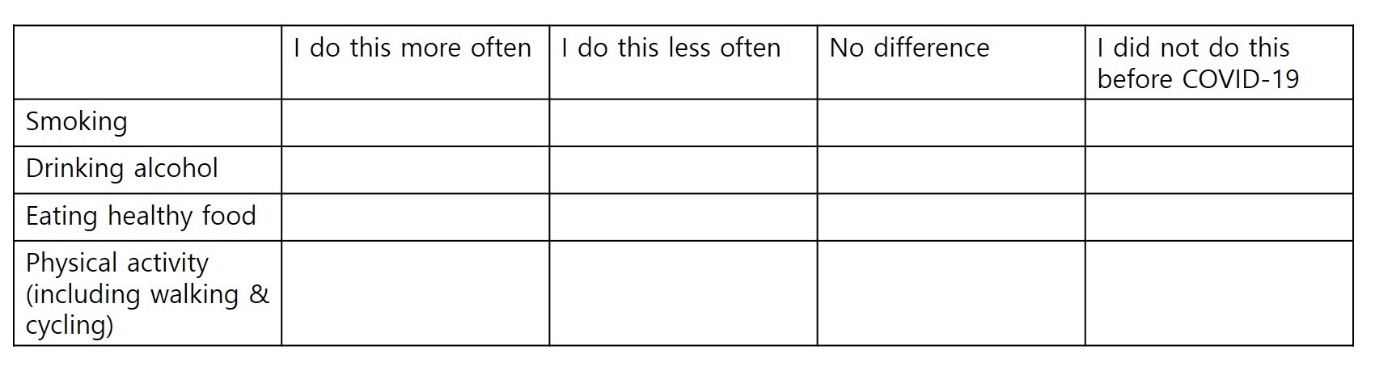 |
| 10. End of survey  Thank you for your time! |

Supplementary Table S2: Baseline characteristics of 121 cohort patients.

| **Characteristics** | **No symptoms (24 months)** | **Symptoms**  **(24 months)** | **Total** | ***P* value** |
| --- | --- | --- | --- | --- |
|  | **(N = 46)** | **(N = 75)** | **(N = 121)** |  |
| Oxygen treatment during acute COVID-19 infection |  |  |  |  |
| Yes | 3 (6.5%) | 12 (16.0%) | 15 (12.4%) | 0.211 |
| No | 43 (93.5%) | 63 (84.0%) | 106 (87.6%) |  |
| ICU admission during acute COVID-19 infection |  |  |  |  |
| Yes | 0 (0.0%) | 4 (5.3%) | 4 (3.3%) | 0.285 |
| No | 46 (100.0%) | 71 (94.7%) | 117 (96.7%) |  |
| MV use during acute COVID-19 infection |  |  |  |  |
| Yes | 0 (0.0%) | 3 (4.0%) | 3 (2.5%) | 0.440 |
| No | 46 (100.0%) | 72 (96.0%) | 118 (97.5%) |  |
| Vaccinated against influenza within the last 6 months |  |  |  |  |
| Yes | 14 (30.4%) | 30 (40.0%) | 44 (36.4%) | 0.386 |
| No | 32 (69.6%) | 45 (60.0%) | 77 (63.6%) |  |
| Newly diagnosed diseases since having COVID-19 infection |  |  |  |  |
| 1. Heart disease | 0 (0.0%) | 1 (1.3%) | 1 (0.8%) | 0.566 |
| 2. Stroke or mini stroke/TIA | 0 (0.0%) | 0 (0.0%) | 0 (0.0%) |  |
| 3. Kidney problems | 0 (0.0%) | 1 (1.3%) | 1 (0.8%) |  |
| 4. Deep vein thrombosis | 1 (0.0%) | 0 (0.0%) | 1 (0.8%) |  |
| 5. Pulmonary embolism | 0 (0.0%) | 0 (0.0%) | 0 (0.0%) |  |
| 6. Other conditions | 3 (6.5%) | 6 (8.0%) | 9 (7.4%) |  |
| 7. None | 42 (91.3%) | 67 (89.3%) | 109 (90.1%) |  |
| Allergic rhinitis |  |  |  | 0.301 |
| Yes | 9 (19.6%) | 22 (29.3%) | 31 (25.6%) |  |
| No | 37 (80.4%) | 53 (70.7%) | 90 (74.4%) |  |
| DM |  |  |  | 1.000 |
| Yes | 7 (15.2%) | 12 (16.0%) | 19 (15.7%) |  |
| No | 39 (84.8%) | 63 (84.0%) | 102 (84.3%) |  |
| HTN |  |  |  | 0.660 |
| Yes | 11 (23.9%) | 22 (29.3%) | 33 (27.3%) |  |
| No | 35 (76.1%) | 53 (70.7%) | 88 (72.7%) |  |
| CKD |  |  |  | 1.000 |
| Yes | 1 (2.2%) | 2 (2.7%) | 3 (2.5%) |  |
| No | 45 (97.8%) | 73 (97.3%) | 118 (97.5%) |  |
| Liver disease |  |  |  | 0.376 |
| Yes | 2 (4.3%) | 8 (10.7%) | 10 (8.3%) |  |
| No | 44 (95.7%) | 67 (89.3%) | 111 (91.7%) |  |
| Solid tumor |  |  |  | 1.000 |
| Yes | 1 (2.2%) | 2 (2.7%) | 3 (2.5%) |  |
| No | 45 (97.8%) | 73 (97.3%) | 118 (97.5%) |  |
| CVA |  |  |  | 0.804 |
| Yes | 1 (2.2%) | 0 (0.0%) | 1 (0.8%) |  |
| No | 45 (97.8%) | 75 (100.0%) | 120 (99.2%) |  |
| COPD |  |  |  | 1.000 |
| Yes | 0 (0.0%) | 1 (1.0%) | 1 (0.8%) |  |
| No | 46 (100.0%) | 74 (98.7%) | 120 (99.2%) |  |
| Heart disease |  |  |  | 0.440 |
| Yes | 0 (0.0%) | 3 (4.0%) | 3 (2.5%) |  |
| No | 46 (100.0%) | 72 (96.0%) | 118 (97.5%) |  |

COVID-19, coronavirus disease 2019; ICU, intensive care unit; MV, mechanical ventilator; TIA, transient ischemic attack; DM, diabetes mellitus; HTN, hypertension; CKD, chronic kidney disease; CVA, cerebrovascular accident; COPD, chronic obstructive pulmonary disease.

Data are presented as n (%).

Supplementary Table S3: Impact of COVID-19 vaccination stratified by number of vaccine doses.

| **Long COVID symptoms (24 months)** | **<3 vaccination doses** | **≥3 vaccination doses** | **Total** | ***P* value** |
| --- | --- | --- | --- | --- |
|  | **(N = 44)** | **(N = 77)** | **(N = 121)** |  |
| Fatigue |  |  |  | 1.000 |
| Yes | 11 (25.0%) | 20 (26.0%) | 31 (25.6%) |  |
| No | 33 (75.0%) | 57 (74.0%) | 90 (74.4%) |  |
| Amnesia |  |  |  | 1.000 |
| Yes | 10 (22.7%) | 18 (23.4%) | 28 (23.1%) |  |
| No | 34 (77.3%) | 59 (76.6%) | 93 (76.9%) |  |
| Insomnia |  |  |  | 0.369 |
| Yes | 6 (13.6%) | 17 (22.1%) | 23 (19.0%) |  |
| No | 38 (86.4%) | 60 (77.9%) | 98 (81.0%) |  |
| Difficulty concentrating |  |  |  | 0.584 |
| Yes | 10 (22.7%) | 13 (16.9%) | 23 (19.0%) |  |
| No | 34 (77.3%) | 64 (83.1%) | 98 (81.0%) |  |
| Depression |  |  |  | 1.000 |
| Yes | 7 (15.9%) | 12 (15.6%) | 19 (15.7%) |  |
| No | 37 (84.1%) | 65 (84.4%) | 102 (84.3%) |  |
| Anxiety |  |  |  | 0.832 |
| Yes | 6 (13.6%) | 13 (16.9%) | 19 (15.7%) |  |
| No | 38 (86.4%) | 64 (83.1%) | 102 (84.3%) |  |
| Cognitive dysfunction |  |  |  | 1.000 |
| Yes | 2 (4.5%) | 4 (5.2%) | 6 (5.0%) |  |
| No | 42 (95.5%) | 73 (94.8%) | 115 (95.0%) |  |
| Anosmia |  |  |  | 0.756 |
| Yes | 2 (4.5%) | 6 (7.8%) | 8 (6.6%) |  |
| No | 42 (95.5%) | 71 (92.2%) | 113 (93.4%) |  |
| Ageusia |  |  |  | 1.000 |
| Yes | 1 (2.3%) | 1 (1.3%) | 2 (1.7%) |  |
| No | 43 (97.7%) | 76 (98.7%) | 119 (98.3%) |  |

COVID-19, coronavirus disease 2019.

Data are presented as n (%).

Supplementary Table S4: Quality of life, PHQ-9, and GAD-7 assessments 24 months following acute COVID-19 infection.

| **Characteristics** | **No symptoms (24 months)**  **(N = 46)** | **Symptoms (24 months)**  **(N = 75)** | **Total**  **(N = 121)** | ***P* value** |
| --- | --- | --- | --- | --- |
| You believe you have completely recovered from COVID-19 |  |  |  | < 0.001 |
| Strongly disagree | 0 (0.0%) | 7 (9.3%) | 7 (5.8%) |  |
| Disagree | 0 (0.0%) | 17 (22.7%) | 17 (14.0%) |  |
| Neither disagree nor agree | 1 (2.2%) | 21 (28.0%) | 22 (18.2%) |  |
| Agree | 14 (30.4%) | 16 (21.3%) | 30 (24.8%) |  |
| Strongly agree | 31 (67.4%) | 14 (18.7%) | 45 (37.2%) |  |
| The healthiest you feel today, on a scale of 0–100; median [IQR] | 90.0  [80.0–90.0] | 80.0  [70.0–85.0] | 80.0  [70.0–90.0] | < 0.001 |
| Intensity of fatigue on average  over the last 24 hours, on a scale from 0–10 where 0 = no fatigue and 10 = worst fatigue; median [IQR]^a^ | 3.0  [2.0–6.0] | 5.0  [2.0–6.0] | 4.0  [2.0–6.0] | 0.089 |
| Extent of breathlessness you feel today (within the last 24 h) |  |  |  | 0.098 |
| Not troubled by breathlessness, except during strenuous exercise | 28 (60.9%) | 31 (41.3%) | 59 (48.8%) |  |
| Short of breath when rushing or walking up a gentle hill | 18 (39.1%) | 39 (52.0%) | 57 (47.1%) |  |
| Walking slower than most people my age because of breathlessness or having to stop for breath when walking at my normal pace | 0 (0.0%) | 3 (4.0%) | 3 (2.5%) |  |
| Stopping for breath after walking 100 yards (90–100 meters) or after walking a few minutes on level ground | 0 (0.0%) | 2 (2.7%) | 2 (1.7%) |  |
| Too breathless to leave the house or breathless when dressing/undressing | 0 (0.0%) | 0 (0.0%) | 0 (0.0%) |  |
| Total PHQ-9 score (depression) |  |  |  | < 0.001 |
| 0–4, None | 45 (100.0%) | 46 (61.3%) | 92 (76.0%) |  |
| 5–9, Mild | 0 (0.0%) | 21 (28.0%) | 21 (17.4%) |  |
| 10–19, Moderate | 0 (0.0%) | 8 (10.7%) | 8 (6.6%) |  |
| 20–27, Severe | 0 (0.0%) | 0 (0.0%) | 0 (0.0%) |  |
| Total GAD-7 score (anxiety) |  |  |  | < 0.001 |
| 0–5, No-to-low risk | 46 (100.0%) | 53 (70.7%) | 99 (81.8%) |  |
| 6–10, Mild | 0 (0.0%) | 19 (25.3%) | 19 (15.7%) |  |
| 11–15, Moderate | 0 (0.0%) | 3 (4.0%) | 3 (2.5%) |  |
| ≥16, Severe | 0 (0.0%) | 0 (0.0%) | 0 (0.0%) |  |

IQR, interquartile range; COVID-19, coronavirus disease 2019; PHQ-9, Korean version of the Patient Health Questionnaire-9 (depression screen for individuals at risk); GAD-7, Generalized Anxiety Disorder-7.

^a^ Intensity of tiredness: 0, none; 2, mild fatigue; 5, moderate fatigue; 10, severe fatigue.

Data are presented as n (%).

Supplementary Table S5: Assessment of cohort patients’ difficulties in performing certain activities because of a health problem.

| **Characteristics** | **No symptom (24 moths)**  **(N = 46)** | **Symptoms (24 months)**  **(N = 75)** | **Total**  **(N = 121)** | ***P* value** |
| --- | --- | --- | --- | --- |
| Difficulty with vision, even when wearing glasses |  |  |  | 0.057 |
| No, no difficulty | 40 (87.0%) | 51 (68.0%) | 91 (75.2%) |  |
| Yes, some difficulty | 5 (10.9%) | 22 (29.3%) | 27 (22.3%) |  |
| Yes, a lot of difficulty | 1 (2.2%) | 2 (2.7%) | 3 (2.5%) |  |
| Cannot do it at all | 0 (0.0%) | 0 (0.0%) | 0 (0.0%) |  |
| Difficulty in hearing, even when using a hearing aid |  |  |  | 0.072 |
| No, no difficulty | 43 (93.5%) | 63 (84.0%) | 106 (87.6%) |  |
| Yes, some difficulty | 2 (4.3%) | 12 (16.0%) | 14 (11.6%) |  |
| Yes, a lot of difficulty | 1 (2.2%) | 0 (0.0%) | 1 (0.8%) |  |
| Cannot do it at all | 0 (0.0%) | 0 (0.0%) | 0 (0.0%) |  |
| Difficulty in walking or climbing steps |  |  |  | 0.006 |
| No, no difficulty | 41 (89.1%) | 48 (64.0%) | 89 (73.6%) |  |
| Yes, some difficulty | 4 (8.7%) | 26 (34.7%) | 30 (24.8%) |  |
| Yes, a lot of difficulty | 1 (2.2%) | 1 (1.3%) | 2 (1.7%) |  |
| Cannot do it at all | 0 (0.0%) | 0 (0.0%) | 0 (0.0%) |  |
| Difficulty in remembering or concentrating |  |  |  | < 0.001 |
| No, no difficulty | 40 (87.0%) | 35 (46.7%) | 75 (62.0%) |  |
| Yes, some difficulty | 6 (13.0%) | 38 (50.7%) | 44 (36.4%) |  |
| Yes, a lot of difficulty | 0 (0.0%) | 2 (2.7%) | 2 (1.7%) |  |
| Cannot do it at all | 0 (0.0%) | 0 (0.0%) | 0 (0.0%) |  |
| Difficulty with self-care like washing all over or dressing |  |  |  | 0.313 |
| No, no difficulty | 44 (95.7%) | 71 (94.7%) | 115 (95.0%) |  |
| Yes, some difficulty | 1 (2.2%) | 4 (5.3%) | 5 (4.1%) |  |
| Yes, a lot of difficulty | 1 (2.2%) | 0 (0.0%) | 1 (0.8%) |  |
| Cannot do it at all | 0 (0.0%) | 0 (0.0%) | 0 (0.0%) |  |
| Using usual (customary) language,  having trouble communicating (for example, in understanding or being understood) |  |  |  | 0.383 |
| No, no difficulty | 43 (93.5%) | 65 (86.7%) | 108 (89.3%) |  |
| Yes, some difficulty | 3 (6.5%) | 10 (13.3%) | 13 (10.7%) |  |
| Yes, a lot of difficulty | 0 (0.0%) | 0 (0.0%) | 0 (0.0%) |  |
| Cannot do it at all | 0 (0.0%) | 0 (0.0%) | 0 (0.0%) |  |

COVID-19, coronavirus disease 2019.

Data are presented as n (%).

Supplementary Table S6: Assessment of lifestyle changes in cohort patients at 24 months after acute COVID-19 infection.

| **Characteristics** | **No symptoms (24 months)**  **(N = 46)** | **Symptoms (24 months)**  **(N = 75)** | **Total**  **(N = 121)** | ***P* value** |
| --- | --- | --- | --- | --- |
| Smoking |  |  |  | 0.008 |
| I do this more often | 1 (2.2%) | 0 (0.0%) | 1 (0.8%) |  |
| I do this less often | 2 (4.3%) | 1 (1.4%) | 3 (2.5%) |  |
| No difference | 7 (15.2%) | 1 (1.4%) | 8 (6.7%) |  |
| I did not do this before or after  COVID-19 infection | 36 (78.3%) | 72 (97.3%) | 108 (90.0%) |  |
| Drinking alcohol |  |  |  | 0.393 |
| I do this more often | 1 (2.2%) | 3 (4.1%) | 4 (3.3%) |  |
| I do this less often | 8 (17.4%) | 13 (17.6%) | 21 (17.5%) |  |
| No difference | 19 (41.3%) | 20 (27.0%) | 39 (32.5%) |  |
| I did not do this before  COVID-19 infection | 18 (39.1%) | 38 (51.4%) | 56 (46.7%) |  |
| Eating healthy food |  |  |  | 0.138 |
| I do this more often | 15 (32.6%) | 36 (48.6%) | 51 (42.5%) |  |
| I do this less often | 1 (2.2%) | 1 (1.4%) | 2 (1.7%) |  |
| No difference | 21 (45.7%) | 19 (25.7%) | 40 (33.3%) |  |
| I did not do this before  COVID-19 infection | 9 (19.6%) | 18 (24.3%) | 27 (22.5%) |  |
| Physical activity  (including walking and cycling) |  |  |  | 0.440 |
| I do this more often | 10 (21.7%) | 26 (35.1%) | 36 (30.0%) |  |
| I do this less often | 10 (21.7%) | 11 (14.9%) | 21 (17.5%) |  |
| No difference | 22 (47.8%) | 31 (41.9%) | 53 (44.2%) |  |
| I did not do this before  COVID-19 infection | 4 (8.7%) | 6 (8.1%) | 10 (8.3%) |  |

COVID-19, coronavirus disease 2019.

Data are presented as n (%).
